# Barriers and facilitators of implementing value-based care: The case of SwissDiabeter

**DOI:** 10.1177/20552076251336322

**Published:** 2025-05-06

**Authors:** Odile-Florence Giger, Elgar Fleisch, Mia Jovanova, Tobias Kowatsch

**Affiliations:** 1 Centre for Digital Health Interventions (CDHI), 595627 Institute of Technology Management, University of St. Gallen, St. Gallen, Switzerland; 2 Department of Management, Technology, and Economics, ETH Zurich, St. Gallen, Switzerland; 3 Centre for Digital Health Interventions (CDHI), 595497 School of Medicine, University of St. Gallen, St. Gallen, Switzerland; 4 Centre for Digital Health Interventions (CDHI), Institute for Implementation Science in Health Care, 27217 University of Zurich, St. Gallen, Switzerland

**Keywords:** Value-based care, qualitative, health financing, diabetes, bundled payments, remote patient monitoring

## Abstract

**Objective:**

Global spending on diabetes care soared to $966 billion in 2021, a 316% surge over the past 15 years. This sharp increase underscores a need for more efficient and cost-effective care strategies. Value-based care (VBC), which prioritizes patient outcomes while controlling expenses, presents a promising solution. However, its real-world implementation remains challenging, particularly in diabetes care. This study examines SwissDiabeter, a proposed diabetes clinic initiative in Switzerland inspired by a Dutch VBC-based Diabeter clinic. We examine key barriers and facilitators during Diabeter's implementation in the Netherlands and assess forthcoming challenges and enablers for SwissDiabeter in Switzerland.

**Methods:**

We employ a deep, extensive embedded single-case design conducting 27 interviews with healthcare professionals, insurers, and patient groups in Switzerland and the Netherlands. The main interview data were complemented by various secondary sources to enhance contextual comprehension, widen perspectives, and validate findings.

**Results:**

We identify four key factors for successful VBC adoption: leadership in driving change, financial restructuring, operational improvements, and enabling digital technologies. We next derive practical recommendations to guide the implementation of value-based diabetes care, redesigning financial incentives for healthcare providers, partnering up with key stakeholders such as insurers or policy makers, and measuring outcomes on a voluntary and anonymous basis.

**Conclusion:**

This study enhances the global discourse on VBC by analyzing key barriers and facilitators in implementing SwissDiabeter, drawing insights from the Diabeter model in the Netherlands. Our findings highlight the need for strong leadership, financial incentives, digital infrastructure, and interdisciplinary collaboration to drive outcome-driven care. Beyond diabetes, these insights provide a framework for scaling VBC across chronic disease management, promoting cost-effective, high-quality healthcare.

## Introduction

More than 537 million adults worldwide live with diabetes.^1^ Poor glucose management is a key factor in the progression of diabetes, leading to both microvascular and macrovascular complications and increased morbidity.^2^ Direct diabetes expenses cost $966 billion in 2021—a staggering 316% global increase over the past 15 years.^1^ Looking ahead, global diabetes rates are expected to double by 2050^3^ with costs estimated to surpass $1054 billion.^4^ Together, these estimates underscore the urgent need for innovative solutions to improve diabetes care and control costs.^4^

In response to this growing crisis, many countries have begun to turn to value-based care (VBC) as a model for chronic disease management.^5^ VBC aims to provide the best outcomes for patients while keeping costs in check. This is often referred to as the “value equation,” where the value is determined by dividing the patient-relevant health outcomes by the cost of achieving those outcomes.^6^ VBC focuses on improving patient outcomes by aligning payment systems with value rather than volume.^7^ In diabetes care, most countries still rely on fee-for-service models, where providers are paid for each service delivered, which can lead to fragmented and costly care.^8^ VBC advocates for outcome-based payment systems, such as bundled payments, where providers receive a single, fixed payment covering all the services needed to manage a patient's diabetes care over time, from regular check-ups to medication. Bundled payments cover the entire care process, encouraging providers to work together efficiently to improve patient outcomes. Unlike fee-for-service, which rewards the number of treatments given, this approach prioritizes quality over quantity.^9^

A pioneering example of VBC in diabetes management is Diabeter, a specialized clinic in the Netherlands. Established in 2006, it applies VBC principles holistically, focusing on comprehensive, patient-centered diabetes care. Its integrated approach leverages six core VBC principles^10^:

1. **Integrated Practice Units (IPUs):** A dedicated team collaborates on personalized diabetes management.
2. **Outcome and Cost Tracking:** Dashboards monitor real-time patient data, improving decision-making.
3. **Bundled Payments:** A single payment covers all diabetes-related care, aligning provider incentives with patient outcomes.
4. **Integrated Care Delivery:** Patients receive streamlined, coordinated care, minimizing inefficiencies.
5. **Scalability:** Diabeter operates across five locations, expanding its impact.
6. **Advanced IT Platforms:** Digital tools facilitate data sharing, remote monitoring, and analytics.

The impact is significant: 55% of Diabeter's pediatric patients maintain HbA1c levels below 7.5% (compared to 28% nationally), with a hospitalization rate of just 3% (versus 8% nationally).^11^ By demonstrating improved health outcomes and cost control, Diabeter sets a benchmark for VBC in diabetes care.^9^

Inspired by Diabeter, SwissDiabeter is Switzerland's first VBC-focused diabetes clinic, aiming to implement a similarly holistic approach. While Switzerland has seen pilot projects incorporating aspects of VBC, no initiative fully integrates all six components outlined by Porter and Lee.^10^ SwissDiabeter seeks to bridge this gap by offering comprehensive, patient-centered care tailored to the Swiss healthcare landscape.

SwissDiabeter will serve insulin-dependent diabetes patients through a multidisciplinary team of diabetologists, psychologists, and nutritionists, with primary hubs in Zurich and Lausanne. To ensure nationwide accessibility, it will leverage telemedicine and real-time glucose monitoring, allowing remote patient engagement. Recognizing the role of family and community support, SwissDiabeter will emphasize patient education and engagement.

This study examines the challenges and opportunities in implementing VBC in Switzerland by addressing two research questions:

1. What are the main barriers and facilitators encountered during the implementation of Diabeter in the Netherlands?
2. What are the expected barriers and facilitators in implementing SwissDiabeter, a VBC-based diabetes clinic in Switzerland?

## Methods

We employed a qualitative deep, extensive embedded single-case design.^12,13^ As a qualitative study, our approach is grounded in an interpretive paradigm, allowing us to explore the complexities of SwissDiabeter in depth. Single-case studies are particularly well-suited for scenarios necessitating deep immersion in the research context, utilization of multiple sources of evidence, and detailed depiction of complex social phenomena within real-world social settings.^14^ This qualitative approach enabled us to capture rich, nuanced insights that would be difficult to achieve through quantitative methods alone, ensuring a comprehensive understanding.

We classified this research as a single-case study because its primary focus was on SwissDiabeter, with the Dutch Diabeter model serving as a contextual reference rather than an independent case. While data were collected in both countries, the unit of analysis remained SwissDiabeter, following an embedded single-case study design where external insights inform, rather than define, the research. Regarding the number of Dutch interviewees, the study prioritized Swiss stakeholders, as the goal was to assess SwissDiabeter's feasibility within the Swiss healthcare system. The Dutch perspectives were included to provide context and best practices, not to create a full comparative study. Expanding the Dutch sample would have shifted the focus toward a dual-country comparison, which was beyond the intended scope.

We primarily utilized qualitative semi-structured interviews with healthcare providers, health insurances, and patient associations as our data collection method. Qualitative interviews captured the intricate aspects of individuals’ lives, including their thoughts, opinions, and perspectives.^15,16^ We used theoretical and purposive sampling logic to identify and select a case that would provide information-rich insights into the perceived barriers and facilitators when implementing a novel VBC clinic in Switzerland.^17^

### Development of the interview guide

We developed our interview guide based on research on innovation research.^18^ First, we identified the prerequisites for using semi-structured interviews and applied our existing knowledge to structure the guide. We conducted a literature review on diabetes management and chronic care management. Additionally, we examined the theoretical foundations of VBC implementation and reviewed grey literature related to diabetes care models, particularly the Diabeter clinic in the Netherlands. To further contextualize our study, we considered insights from health system comparisons between Switzerland and the Netherlands.

Second, we formulated a preliminary semi-structured interview guide consisting of two levels: main themes and follow-up questions. Each participant was asked about core themes, while pre-designed and spontaneous follow-up questions allowed for deeper exploration of specific topics.^19^

Third, we pilot-tested the guide with a healthcare expert outside the research team to assess its clarity, appropriateness, and comprehensiveness. This led to refinements in the wording and structure of certain questions.^20^

During the data collection phase, the interview guide was iteratively adapted as new insights emerged, enabling us to refine our focus on key barriers and facilitators influencing the implementation of SwissDiabeter in the Swiss healthcare system.

### Data collection

Consistent with methodological single-case study guidelines, we leveraged diverse data sources: interviews (primary data) and news articles, marketing and communications materials and internal documents (secondary data) (see Table 1).^13–21^ Consistent with guidelines for exploratory and case-oriented research, the main interview data were complemented by various secondary sources to enhance contextual comprehension, widen perspectives, and validate findings.^13,14^

**Table 1.** Overview of data sources.

	**Primary data** Interviews	**Secondary data**		
		News articles and opinion pieces	Marketing and communications materials	Internal documents
**Data items**	27	22	8	5
**Time of data collection**	April, 2024–August, 2024	May, 2024–July, 2024	May, 2024–July, 2024	May, 2024–July, 2024
**Description of the data**	Semi-structured interviews with nine healthcare providers, nine insurances, and five patient associations conducted via video call and in person Semi-structured interviews with founders and management executives and insurances of Diabeter in the Netherlands conducted via video call	Relevant news articles of relevant stakeholderss (e.g. insurances, healthcare providers)	Product and service information published online and print of relevant stakeholders (e.g. insurances, healthcare providers)	Internal information, documentation and organizational structure articles
**Role in analysis**	Interviews: Overview of the barriers and facilitator when implementing a VBC clinic and derive recommendations for action	Descriptions of the company's strategic and operational endeavors concerning VBC aspects. Information around new services and views on VBC	Demonstrations of the services including VBC.	Descriptions of internal structure and processes concerning VBC aspects.

We conducted 27 in-depth interviews between April and August 2024 with twelve healthcare providers, ten payers, five patient associations from Switzerland, three healthcare providers from Diabeter Netherlands, and one health insurer from the Netherlands (see Table A1 in Appendix C for interviewees' backgrounds).

Each interview followed an open-ended and semi-structured format, enabling participants to address significant topics. This approach also allowed researchers to delve deeper into areas requiring clarification.^22^

The interviews started with participants introducing themselves personally and receiving an overview of the study's framework (see Appendix A for the interview templates). Sociodemographic inquiries are succeeded by discussions regarding possible barriers and facilitators of implementing SwissDiabeter. Toward the conclusion, participants were encouraged to pose additional questions or share any thoughts about barriers and facilitators that had not been addressed. We recruited the participants through purposeful sampling. Moreover, we used the snowballing technique to obtain further perspectives. All the participants spoke German or English.

Most of the interviews were conducted by the lead researcher (OFG) and the rest was done by two research assistants, ensuring consistency in questioning and the exploration of key themes related to the implementation of SwissDiabeter. Saturation was achieved when no new themes emerged from the data. Data collection continued until additional interviews no longer provided novel insights, indicating that key patterns and themes had been fully explored.

This study was approved by the institution's ethics committee and classified as ethically unproblematic, exempting it from formal evaluation. Prior to conducting the interviews, participants were fully informed about the study's purpose and procedures. Written consent was obtained for their participation, including permission to audio record the sessions. Participants were assured of their right to withdraw from the study at any time. Furthermore, all data collected was anonymized and securely stored to ensure confidentiality. We adhered to the COREQ (Consolidated Criteria for Reporting Qualitative Research) guidelines throughout the study to ensure rigor and transparency (see Appendix B).^23^

### Data analysis

The interviews were digitally recorded and transcribed using the software Dovetail^24^ yielding 304 pages of interview transcripts. The transcripts were reviewed to ensure accuracy, were anonymized, and given unique study IDs. Data collection and analysis were conducted simultaneously, utilizing ATLAS.ti software.

The data analysis followed an inductive thematic coding approach. Initially, the lead researcher (OFG) and two research assistants conducted open coding to identify key themes and patterns without a predefined coding framework, allowing themes to emerge directly from the data.^25^ To ensure inter-coder reliability, the researchers first coded a subset of transcripts independently and then discussed their codes to assess consistency. Based on this discussion, the codebook was refined iteratively, resolving discrepancies through consensus. As themes developed, axial coding was applied to group related concepts and establish relationships between categories. Although the initial coding was purely inductive, the final framework was aligned with existing literature to contextualize findings. This structured approach ensured inter-coder reliability, strengthened validity, and facilitated a rigorous, iterative integration of emerging themes.

**Figure 1.**
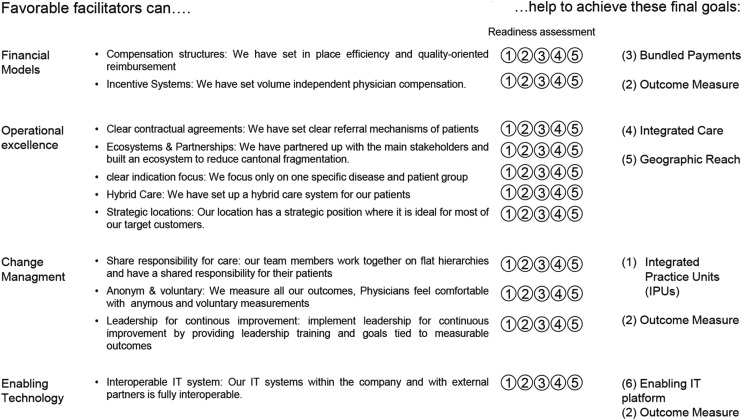
Readiness assessment framework for VBC.

Facilitators were matched to barriers by aligning expert-identified challenges with their proposed solutions. For example, “market scalability concerns” were paired with “including obesity treatments” to broaden the market. Where experts did not provide direct matches, the author team matched barriers and facilitators. The final step involved interviews with a leading Swiss health insurance, which helped identify four aggregated dimensions (e.g. financial models, operational excellence, change management, enabling technology) to structure the framework (see Figure 1 in the discussion). The coding structure is depicted in Table 2.

**Table 2.** Coding structure.

Categories	Themes	Subthemes	
		Barriers	Facilitators
**Financial Models**	Scalability	Market scalability concerns (CH)	Including obesity treatments (CH)
	Telemedicine and on-site locations	Reluctance toward telemedicine and travel time (CH)	Political engagement (CH) Monetary Incentives (CH) Education (CH) Local treatment networks (CH)
	Incentives	No incentives (NL)	Self-employed physicians (NL)
	Bundled payments	Challenges of bundled payments (CH)	Single-case bundled payments (CH)
	Financials	Financial uncertainty (CH)	Pilot projects (CH)
**Operational excellence**	Fragmentation	Fragmented cantons and regulations (CH)	
	Partners vs competitors	Fear of losing patients (NL) Fear of losing patients and research opportunities (CH)	Partner to provide education (NL) Building partnerships (CH)
**Change Management**	Hierarchies and measurements	Fear of measurements (CH and NL)	Flat hierarchies (NL) No finger pointing (NL) Extra resources (NL) Hospital-level comparisons (CH)
**Enabling Technology**	IT system	Fragmented IT systems (NL)	Interoperable IT-system (NL and CH)

In addition to interviews, this study analyzed secondary data, including news articles, marketing materials, and internal documents, to triangulate findings and validate key themes. Using a qualitative approach, secondary data were coded inductively with ATLAS.ti and analyzed alongside interview data to identify contextual insights. Cross-referencing these sources allowed for a deeper understanding of SwissDiabeter's planned implementation and Diabeter's established model in the Netherlands. These secondary sources validated key findings by confirming trends in barriers, facilitators, and implementation strategies. They enriched the analysis by offering contextual depth beyond interview responses, strengthening the study's conclusions.

## Results

Before presenting the identified barriers and facilitators, it is essential to outline the core principles of the VBC framework, which serve as the foundation for both Diabeter in the Netherlands and SwissDiabeter in Switzerland. Table 3 provides a structured overview of the six fundamental VBC principles, detailing how they have been applied in Diabeter and how SwissDiabeter aims to adopt and adapt them to the Swiss healthcare system. This comparison helps contextualize the challenges and opportunities for VBC implementation in Switzerland by highlighting structural differences and strategic adaptations between the two models.

**Table 3.** Overview VBC and its application.

VBC principle	Definition	Application at Diabeter (NL)	Planned application at SwissDiabeter (CH)
**Integrated Practice Units (IPUs)**	Multidisciplinary teams organized around the patient's condition^10^	Diabetes specialists, nurses, and dietitians work together in a single unit	SwissDiabeter will have a team of endocrinologists, nutritionists, and psychologists in a SwissDiabeter hub in Zurich and Lausanne
**Measuring Outcomes and Costs**	Tracking patient-relevant health outcomes and cost-effectiveness^10^	Continuous glucose monitoring, tracking HbA1c levels, and other measures and cost per patient outcome	SwissDiabeter will integrate telemedicine to track real-time outcomes
**Bundled Payments**	Single payment for entire care cycle instead of fee-for-service^10^	Insurers pay a fixed amount covering all diabetes-related services	Pilot projects for bundled payments to be tested in Switzerland
**Integrated Care Delivery**	Seamless coordination across providers^10^	Diabeter partners with hospitals for complex cases	SwissDiabeter might collaborate with local GPs and specialists
**Geographic Expansion**	Scaling care delivery to multiple locations^10^	Diabeter operates five clinics in the Netherlands	SwissDiabeter will start with two hubs (Lausanne and Zurich) and expand via telemedicine
**IT-Enabled Care Platforms**	Using digital tools for patient management and data sharing^10^	Diabeter's proprietary disease management system integrating patient data	SwissDiabeter will implement telemedicine and interoperable IT systems

## Barriers

### Financial models

#### Theme—scalability

*Subtheme: Market scalability concerns (CH):* The potential market for specialized diabetes care may be perceived as too small to justify extensive investments and scalable operations, limiting the clinic's growth and reach (Interviewees 11, 18, 19, 20, and 22). Also, only around 20% of patients use connected diabetes pump systems, which are required to continuously track patients’ health data (Interviewee 1):

*“But then it’s also a question of quantity, in the sense of how many are there? And there are not many. Type 1 diabetics about 0.5%.” (Interviewee 20)*

#### Theme—telemedicine and on-site locations

*Subtheme: Reluctance toward telemedicine and travel time (CH):* Patients and healthcare providers may hesitate to adopt telemedicine and remote monitoring technologies because of concerns over the privacy and security of sensitive health data. This reluctance can stem from fears of data breaches, unauthorized access, and misuse of personal health information. Furthermore, it was mentioned that patients, especially older adults, do not want to use telemedicine and prefer to meet their doctor in person (Interviewees 2, 5, 6, 16). This was mentioned mainly from the healthcare providers perspective. Nevertheless, many interviewees from the patient's association perspective did not see telemedicine and remote monitoring as barriers; many also mentioned the opportunities. Others raised the concern that patients feel uncomfortable being monitored constantly, as they might feel that their health data is something intimate (Interviewees 3 and 11).

*“The advantage is certainly that you can do certain things remotely. You don’t always have to meet up. There are also disadvantages. There are patients who appreciate direct contact and don’t feel comfortable doing it remotely. Overall, the advantages outweigh the disadvantages. But there are also situations where face-to-face contact is more effective.” (Interviewee 6)*

*“The other is data protection. We now have a good lawyer again. Before, it was a bit complicated that you couldn’t implement such things. And not that 20 people are suddenly listening. And maybe the employers, too.” (Interviewee 3)*

Implementing telemedicine and remote monitoring involves administrative hurdles, including tariff system adjustments and regulatory compliance, which can be challenging to navigate. Telemedicine consultations cannot be billed with as many TARMED points (Swiss health tariff system) as on-site consultations. This makes it difficult to be profitable with telemedicine (Interviewee 1, 2, 3, 9):

*“The so-called office hours, when you still have to deal with paperwork etc., you also get paid, but it’s significantly less. And that means that as a manager, you always have to make sure that your employees see as many patients as possible during their working hours.” (Interviewee 2)*

*“Then comes the next question: how do you bill for it? The diabetes nurse who does this has a certain amount of time. This is still a difficulty in telemedicine. How can I bill for it? That's possible, but you will not earn as much as with on-site consultations.” (Interviewee 3)*

The requirement for patients to travel to one of the two SwissDiabeter clinics can be a significant burden, especially for those living far away or with limited mobility. Interviewees mentioned that in Switzerland, individuals are not used to travel long distances to get medical care. Therefore, they would not accept to travel more than 30 minutes to get to the doctor. Also, if they move to another place within Switzerland, they usually do not stay with the same doctor. Instead, they change to a doctor where they live close by (Interviewee 1, 2, 3, 5, 11, 12):

*“especially when people live a little bit further away, maybe don’t always have a car or can’t drive, that’s the point. Then they prefer to be close and they don’t want to drive 45 minutes or an hour to get there.” (Interviewee 12)*

#### Theme—incentives

*Subtheme: no incentives (NL):* It was mentioned that physicians were used to the old fee-for-service system. When Diabeter introduced outcome-based compensation, doctors were more willing to support new VBC approaches. This was mentioned by Interviewee 7:

* “the hospital is a big cake. And if you want a bigger size, I get a smaller one. It’s a competitive surrounding where nothing is about outcome. And that’s what we learned in the hospitals the hard way, but also wanted to use in our clinic in the right way.”*

#### Theme—bundled payments

*Subtheme: Challenges of bundled payments (CH):* The transition to bundled payment systems, where a single payment covers all services related to a treatment episode, can be complex and challenging. This financial model requires significant restructuring and coordination among providers. Especially in the field of diabetes, where many different disciplines (endocrinology, nutritionists, ophthalmologist, etc.) are involved, it might be hard to calculate the bundle for a patient correctly. Furthermore, it will be challenging to make a cut, what treatment is still in the bundle and what not, in case there are complications (Interviewees 16, 17, 18, 19, 20).

*“The challenge is to agree on a flat rate that balances both sides’ interests. We want to ensure the rate isn’t set too high, as that would lead to overpaying and put strain on basic insurance. On the other hand, the service provider wants to avoid a rate that’s too low, as it could make it difficult for them to stay financially viable in the long run. […].” (Interview 18)*

*“What are diabetes-related costs and what are other costs?” (Interview 20)*

#### Theme—financials

*Subtheme: Financial uncertainty (CH):* Implementing SwissDiabeter on a large scale is a complex and resource-intensive project. The significant time and financial investment required raise questions about funding and sustainability (Interviewees 13, 15, 18, and 19):

*“Then, of course, there is the question of economics. Who would have the incentive to initiate such a structure, i.e., to invest the capital? It’s not cheap. And then, and I think this is also a crucial point, can you somehow amortize such a structure with the size of the market in Switzerland?” (Interviewee 18)*

### Operational excellence

#### Theme—fragmentation

*Subtheme: Fragmented cantons and regulations (CH):* It was mentioned that there is cantonal fragmentation within Switzerland that poses a major barrier; for example, not all cantons allow telemedicine models and sometimes if some treatments are done in a different canton than where the patient lives, it need to be justified (Interviewee 2, 5). Also, healthcare providers have different processes for different patients. There are no standardized processes (Interviewee 3).

#### Theme—partners vs competitors

*Subtheme: Fear of losing patients (NL):* When Diabeter was first introduced, healthcare providers in other general hospitals feared losing their patients to the new Diabeter clinic. This concern was highlighted by Interviewee 8:

*“What we didn’t expect was the reaction of the colleagues in other hospitals. We were almost literally excommunicated from the pediatric society. Other hospitals were so afraid that they were losing their patients that they wrote letters to patients and to family practitioners to say, we were a dangerous initiative where you should never go to, and if you would go to Diabeter, we would never accept you back in the regular hospital.”*

*Subtheme: Fear of losing patients and research opportunities (CH):* In Switzerland general practitioners and hospitals may worry about losing patients to specialized clinics like SwissDiabeter, which could diminish their patient base and involvement in diabetes research (Interviews 2, 3, and 4). In particular, some healthcare providers express skepticism about participating in or co-building initiatives like SwissDiabeter. In-patient facilities fear losing access to patients who require overnight medical services, which may lead to downsizing decisions driven by concerns about revenue loss. This situation can result in a reduced influence and budget for their departments, particularly in the case of endocrinology clinics. Chief medical officers often have their salaries tied to the revenue generated by their sub-clinics, so transferring patients to specialized clinics could diminish their financial standing and influence within the hospital. Additionally, executives at large hospitals may prioritize other departments, such as cardiology or oncology, over innovative diabetes management approaches (Interviewee 2).

*“Because people are all cared for somewhere, and every patient that would be discharged there will be taken away from someone, and they will fight back. Experience just shows that. […]. All services that we do not remunerate are factored into someone’s income and that is why there is resistance.” (Interviewee 2)*

### Change management

#### Theme—hierarchies and measurements

*Subtheme: Fear of measurements (CH & NL):* When Diabeter started, some healthcare staff were uneasy about being measured and compared to other teams since they weren’t used to this system. Additionally, tracking and reporting patient outcomes took a lot of their time. This was mentioned by Interviewee 21:

*“When we started the doctors were not so happy about it because they said we are doing the best in the world so why do we have to measure our results but afterwards they saw that they could learn from other hospitals other medical specialists and when they saw that they were really enthusiastic about the process and the very best healthcare […].” (Interviewee 21)*

In Switzerland, physicians may fear being evaluated and compared based on their performance, leading to resistance to adopting new systems that facilitate such assessments. Concerns about job security and reputation can also hinder cooperation (Interviewees 4, 5, and 23):

*“That is an interesting approach but harbors some potential for conflict, as is always the case with ratings and comparisons. I always find it difficult to measure the performance of a diabetologist by a few key figures.” (Interviewee 5)*

### Enabling technology

#### Theme: IT systems

*Subtheme: Fragmented IT systems (NL & CH):* One of the significant challenges in implementing the Diabeter clinic was the fragmentation of IT systems across the healthcare sector in the Netherlands. Different healthcare providers, hospitals, and insurance companies often used disparate and incompatible IT systems.

“*Another example is fragmentation. I mean, technology is key to improvement. And if you work in a hospital and your IT department says, we cannot integrate this program in our system, you’re lost. If you cannot get the data, you’re lost. There are nice programs where the patient can upload data, but hospitals can’t access that because of hospital rules.”* (Interviewee 8)

In Switzerland, providers noted that while many IT systems exist, they cannot be integrated with other systems within Switzerland. For SwissDiabeter, developing a new IT system would only be useful if it can work seamlessly with other systems, including those used by general practitioners (Interviewees 2, 3, and 6).

## Facilitators

### Financial models

#### Theme—scalability

*Sub-theme: Including obesity treatments (CH):* Extending services to cover obesity management can broaden the clinic's target market and enhance scalability. Addressing related health issues increases the clinic's relevance and patient base (Interviewee 11):

*“In Switzerland, type 2 is said to be around 500,000 and of those around 10%, then you get around 40,000. They certainly don’t all come to Zurich. So, you would probably have to expand that a bit. But what you can think about, of course, is a moderate obesity center and diabetes. Obesity is simply the future.” (Interviewee 11)*

From an insurance perspective, it was mentioned that expanding to other diseases where the patient is not on medication is financially less attractive. Health insurers aim to maintain a balance of healthy individuals and chronically ill patients, provided the latter are covered by Switzerland's risk equalization mechanism (“Risikoausgleich”). Patients whose chronic conditions are well-managed help mitigate cost risks. Insurers benefit from ensuring that these patients receive proper management, as this minimizes the likelihood of expensive complications. On the other hand, poorly managed chronic patients pose a significant financial risk due to increased healthcare costs. Therefore, insurers are incentivized to focus on maintaining a mix of healthy individuals and well-managed chronically ill patients, as this balance is the key to financial stability. Patients with conditions that do not trigger additional payments through the risk equalization mechanism (e.g. obesity) are less financially attractive (Interviewee 27).

#### Theme—telemedicine and on-site locations (CH)

*Subtheme: Political engagement (CH):* Actively engaging with policymakers to advocate for changes in the tariff systems can facilitate the integration of telemedicine and remote monitoring services. Political support can streamline administrative processes and regulatory adjustments (Interviewees 3 and 9).

*“Now comes the TARDOC. There you can look again to see if there is a fairer distribution of TARMED points. Ideally, there is someone who engages actively in the policy-making system.” (Interviewee 3)*

*Subtheme: Monetary incentives (CH):* Financial incentives can motivate patients and healthcare providers to use the new system. Monetary rewards can encourage participation and adherence to new protocols and technologies. For example, patients can get a lower premium when they go to SwissDiabeter or voucher points when they manage their condition well (Interviewees 16 and 19).

The other, of course, is incentives. So there is a possibility, for example, that service providers or even patients get a discount if they are well-adjusted. Let me give you an example of dental health. Dental health in Switzerland is different from that in Germany. So if you have well-maintained teeth and you get something like a well-maintained car, then you should actually get a benefit from it in principle, right? So, people who are in good shape must be incentivized somehow (Interviewee 16).

*Subtheme: Education (CH):* Providing comprehensive education and training to patients and healthcare providers can facilitate the adoption of new practices and technologies. This includes teaching them about the benefits, usage, and safety measures of telemedicine and remote monitoring (Interviewees 6 and 9):

*“Staff need to be adequately trained in the various areas. A high degree of independence. Technical training is increasingly important. And the prerequisites that all people working in the individual areas must have.” (Interviewee 6)*

*Subtheme: Local treatment networks (CH):* Developing networks of local healthcare providers who can treat patients on-site can alleviate the need for long-distance travel. This approach ensures that patients receive care within their communities, improving accessibility and convenience (Interviewees 16, 17, and 9).

*“But as I said, it would probably be very good for SwissDiabeter to join forces with a network organization. There are various networks of doctors in primary care in Switzerland. And they are now quite large. One of the most famous is Medics.” (Interviewee 17)*

*“More outpatient, specialized services are needed. That is the future.” (Interviewee 9)*

#### Theme—incentives

*Subtheme: Self-employed physicians (NL):* Interviewee 21 highlighted how financial structures can shape incentives, noting that self-employed doctors within a clinic may be more motivated to align with the clinic’s broader goals, such as improving patient outcomes:

*“in Holland a part of the medical staff of the hospital has their own company. You know, they are not on the payroll of the hospital. If you still pay for fee for service to the medical specialist, they keep focusing on volume instead of outcomes.”*

#### Theme—bundled payments

*Subtheme: Single-case bundled payments (CH):* Initiating bundled payment systems with single-case implementations can help manage complexity and provide a controlled environment for testing and refinement. This phased approach allows for gradual adaptation and troubleshooting.

*“And then, after a period of time, you look at what the average cost is there. Then you also have a basis to say at the end, okay, now we know what they cost on average. And then you go to a bundled payment, which is easier for everybody.” (Interview 19)*

#### Theme—Financials

*Subtheme: Pilot projects (CH):* Starting with small-scale pilot projects can help manage risks and refine the implementation process. Involving patients and healthcare staff in co-creating the project can identify potential issues early and ensure that the system meets the needs of all stakeholders (Interviewees 13, 14, and 15):

*“But definitely try to show in the small sandbox prototype that it can work and somehow continue step by step.” (Interviewee 15)*

### Operational excellence

#### Theme—partner versus competitors

*Subtheme: Partner to provide education (NL):* Diabeter began forming partnerships with hospitals to provide guidance and education for diabetologists, thereby enhancing their knowledge and supporting research efforts within the clinics. This strategy was mentioned by Interviewee 7:

*“we do have some links with the hospitals. If a patient is admitted, we can, in certain circumstances, go for consultation to that hospital to bring in the knowledge. They are the main physicians, so we only are in an advisory role. Other relationship is related to research, let’s say, where we need to have a relationship with an expert genetic lab or biochemistry lab or something like that, but it’s really more on the purposeful research projects.”*

*Subtheme: Building partnerships (CH):* Forming strategic partnerships with hospitals, independent diabetologists, and GPs can integrate SwissDiabeter into the broader healthcare ecosystem. Collaborative efforts can ensure shared patient care and ongoing research participation. For example, easier cases can be transferred from the hospital to SwissDiabeter, whereas more complex cases will be transferred from SwissDiabeter to the hospitals. Instead of collaborating with a large university hospital from the start, it might be beneficial to build up SwissDiabeter independently, aiming to achieve outstanding results that could later convince hospitals to join as collaborators (Interviewee 21). Also, some experts mentioned that building up a network within Switzerland, including GPs and diabetologists, could improve collaboration between these stakeholders and make sure patients do not need to travel so long (Interviewees 17, 19)

*“So I see it more like they planned one or two centers, probably more would be needed. So smaller, but more spread out. […]. So the alternative to this clinic Diabeter would be a network.” (Interviewee 17)*

### Change management

#### Theme—hierarchies and measurements

*Subtheme: Flat hierarchies (NL):* According to the founders of Diabeter, it is crucial to implement flat hierarchies within the teams and establish a culture of continuous learning. Coming regularly together at the same coffee machine regardless of the hierarchy. Also, every 6 months, Diabeter had workshops on improving processes and workflows where the entire staff participated, not only the management executives.

*“So there is no other corridor or room for different employees. They have the same coffee machine; you have the same work location. In the back office, they have a separate consultation room, but they are close by and near each other to enhance collaboration.” (Interviewee 7)*

*“Over the years, we observed that the leading medical specialist became increasingly proud of the results. And we gave them the opportunity to speak about the results in the media, in the newspapers or during meetings.” (Interviewee 21)*

#### Subtheme: no finger-pointing (NL)

Quality and outcome measures must be established slowly, and participation should be non-mandatory. Also, it is important that employees can participate anonymously. This helps the staff to not feel finger-pointed and accused. This was mentioned by Interviewee 7:

*“I introduced it first in an anonymized way. So I honored one of those Diabeter days. The staff was just as an entertainment after lunch, bowling, and they were looking to each other. And they looked to each other how they did it. They helped each other. And so I watched that, and that was my introduction on the performance, let’s say metrics that I said when I looked at you, you were really trying to perform and to learn. But the strange thing is you do that with bowling, but you don’t do that with patient care. So I then told them anonymously the metrics. It was a kind of, wow, oh, how good or how bad is that? How can we change that? And as I said, every one of you can make an appointment with me, and then you get your numbers and we can go through your patient list and I give advice, what you might change. It’s not that you do wrong, but there might be 10% of your patients where you can do something.”*

#### Subtheme: extra resources (NL)

In the beginning, when measuring processes are not yet established, hiring extra staff to help with these tasks is important. Also, it is crucial to educate the staff on the easiest way to collect new data. Also, automate the measurements early on with the help of technology and allow employees more time for some processes.

*“The third lesson learned is to give them the support they need because collecting the data is a lot of work, and they have to do a lot of registration.” (Interviewee 21)*

*Subtheme: Hospital-level comparisons (CH):* Performance comparisons should be made at the hospital rather than individual level to mitigate physicians’ fears. This approach may foster a collaborative environment and reduce pressure on individual healthcare providers (Interviewees 17 and 19):

*“I would like to have a learning system between these clinics. If doctors are smart, then they measure their quality internally and then see who does what better in order to learn from each other, not to somehow create a ranking list.” (Interviewee 17)*

### Enabling technology

#### Theme—IT systems

*Subtheme: Interoperable IT system (NL & CH):* From the start, Diabeter built an advanced, interoperable IT system that became the foundation of its operations. This system integrates patient data, streamlines communication between healthcare providers, enhances diabetes care, and enables the measurement of patient outcomes. The founders consider this one of the most important success factors for implementing it right from the beginning. Also, it was mentioned that it can be crucial to invest time and effort in creating interoperable systems across different clinics that are important for you as a hospital. Although it takes a lot of time, the benefit will exceed the effort invested (Interviewee 21).

*“So everyone uses the same system. Everyone adheres to the Vcare system. That is the EMR. It’s not a simple EMR, it’s a smart EMR. So it has dashboards, it has integrated protocols and pathways, so you know what to do. You see for example when do you need to take your cholesterol, lipid test, etcetera. And also I built prescription algorithms in the system. So now at least every patient is on a minimal, fair and good setting of your pump settings. So everything is analysed by an algorithm built upon the best performing patients and theoretical approach. And that makes it also easy, because where it takes otherwise a long time of preparation and calculations, it’s now analysed in 30 seconds. And it synchronizes also to the patient at home in the patient app.” (Interviewee 7)*

Also, in the case of Switzerland, it was mentioned that the adoption of international interoperability standards can facilitate better data exchange between disparate systems. Encouraging healthcare providers to implement these standards can improve the seamless flow of information.

*“What we really need are interoperable systems that are standard-based. So everyone can connect.” (Interviewee 27)*

## Discussion and recommendations

There were many overlaps in the barriers and facilitators when comparing the Netherlands and Switzerland. Nevertheless, some were only mentioned in Switzerland. For example, reluctance toward telemedicine and remote monitoring due to data security concerns and the time-consuming travels to SwissDiabeter for patients as there will be only two hubs in Switzerland (Zurich and Lausanne).^26^

Another challenge unique to Switzerland was the issue of geographic accessibility. Unlike the Netherlands, where healthcare services are relatively evenly distributed due to high population density, Switzerland faces logistical constraints in scaling centralized diabetes care hubs. SwissDiabeter plans to establish only two main centers (Zurich and Lausanne), which may result in increased travel burdens for patients in more remote areas. Prior literature emphasizes that geographical disparities significantly impact healthcare access in Switzerland, particularly in rural and mountainous regions, where specialist care is often limited.^27^

From a market scalability perspective, it was noted that Switzerland's lower population density could make the business case for SwissDiabeter less attractive. The Netherlands ranks second in Europe in terms of population density (512 individuals per square kilometer), whereas Switzerland has less than half that density, with 218 individuals per square kilometer.^28^

However, this lower density may paradoxically support the case for telemedicine and remote monitoring solutions, particularly in Switzerland's lowland and mountainous regions, where healthcare access remains challenging.^29^ Studies suggest that telemedicine can significantly improve healthcare delivery in remote Swiss communities, reducing unnecessary hospital visits and increasing continuity of care.^29,30^ Therefore, leveraging digital health infrastructure in SwissDiabeter could help mitigate accessibility concerns, particularly for patients requiring specialist input beyond their local general practitioners.

Additionally, the financial feasibility of digital diabetes management programs in Switzerland remains a debated issue. While the Netherlands has implemented VBC models that facilitate reimbursement for telemedicine-driven interventions, the Swiss reimbursement system is still evolving. Future policy adjustments, such as incorporating bundled payments for chronic disease management, could enhance the economic viability of SwissDiabeter and similar initiatives.

### Recommendations

The barriers and facilitators were explored in interviews with a leading Swiss health insurer, resulting in a refined framework (see Figure 1) to support the launch of a Swiss VBC diabetes clinic in 2025. This framework aligns key facilitators with VBC components and represents critical strategies for implementing VBC. The facilitators, identified through expert interviews, are categorized into four domains: Change Leadership, Financial Models, Operational Excellence and Enabling Technology. Each facilitator was mapped to one or more of the VBC components to illustrate how specific organizational changes can lead to achieving key VBC goals. For instance: Facilitators under Change Leadership support the development of Integrated Practice Units (IPUs) and the creation of robust outcome measures.

This framework helps VBC projects evaluate their maturity, highlighting progress and identifying gaps for improvement. The 1 to 5 readiness scale assesses an organization's preparedness to implement key facilitators, providing a structured approach to align processes with the six VBC components and guide targeted interventions for achieving VBC goals.

### Change Management

#### Barriers

Lack of shared responsibility and siloed leadership structures are common barriers to implementing change. Flat hierarchies and teamwork between staff and leadership are often missing, which reduces collaboration. Another challenge is the discomfort among physicians with transparent outcome measurement, particularly when there are concerns about being evaluated based on these results. Moreover, leadership for continuous improvement is often not clearly defined, and there may be resistance to setting measurable outcomes.

#### Facilitators (if not yet ready)

To overcome barriers in change leadership, organizations must start by cultivating a **culture of shared responsibility** for patient care. This can be achieved through leadership development programs that focus on collaborative, team-based care models. Leaders should be trained to facilitate flat hierarchies, where decisions are made collaboratively, encouraging every team member to take ownership of patient outcomes. Although it has been shown that establishing flat hierarchies are difficult to achieve, some studies showed that it can be negotiated or challenged through various strategies, such as assertive leadership, collaborative communication, and structured feedback.^31^

Introducing **anonymous or voluntary outcome measurement** systems is a critical first step to alleviate the fear of scrutiny among physicians. By ensuring that the initial measurement processes are non-punitive, healthcare teams will gradually build trust in the system. Simultaneously, leadership training should emphasize the importance of **continuous improvement** through measurable goals. Leaders should be equipped with skills to set specific, measurable, achievable, relevant, and time-bound (SMART) goals linked to outcomes. Also, studies mention that creating a new position with a Chief medical officer that is responsible for change management is important.^32^ Here, specific training sessions or symposiums explaining the aims of VBC could further help in this regard.^33^

### Financial models

#### Barriers

The absence of efficient compensation structures and incentive systems aligned with VBC principles is a significant barrier. Fee-for-service models, which reward quantity over quality, do not promote efficiency or improved outcomes. Physicians often lack the incentives to focus on value-driven care, and reimbursement models may not account for bundled payments across care cycles, making it difficult to shift toward a VBC framework.

#### Facilitators (if not yet ready)

Transitioning toward VBC requires restructuring financial models. One of the first actions is to introduce **bundled payments**, where payments are made for the entire care cycle, rather than individual services. This encourages efficiency and collaboration across departments, as the entire care team shares responsibility for both cost management and outcomes.

To support this shift, organizations should design **compensation structures** that reward quality over quantity. For example, volume-independent physician compensation ensures that physicians are incentivized to deliver high-quality care rather than focusing on patient turnover. A study comparing Kaiser Permanente's integrated care model and capitation system with Germany's fee-for-service health system supports this idea. The study found that Kaiser Permanente, which incentivizes efficiency and quality, achieved better patient outcomes than the German system.^34^ Although, a flat-rate fee is not yet standard for chronic conditions, it has been proposed in other areas like ambulatory surgery in Switzerland (Interviewee 27).

**Financial incentives** should be tied to outcome metrics, encouraging healthcare professionals to deliver VBC that improves patient outcomes while reducing costs. This approach would encourage doctors to prioritize the quality of care over the quantity of patients treated. A study supports this by indicating that the introduction of Pay-for-Performance and bundled payment programs generally leads to improved patient outcomes.^9^

The importance of adapting incentives for physicians was also highlighted at the Blue Cross Shield of Massachusetts, which rewarded physicians for quality improvement and cost management.^35^

### Operational excellence

#### Barriers

Operational excellence requires well-established processes, partnerships, and patient care pathways. However, organizations often struggle with fragmented systems, unclear referral mechanisms, and the absence of strong partnerships with key stakeholders. This lack of coordination increases inefficiency and limits the ability to provide integrated care across separate facilities. Additionally, many organizations lack focus on specific disease groups or geographic reach, which hinders their ability to scale VBC.

#### Facilitators (if not yet ready)

Achieving operational excellence starts with creating clear, standardized processes across the organization.

**Building up ecosystems** is crucial through strategic partnerships with key stakeholders, such as hospitals, insurers, and primary care providers.^36^ These collaborations help to overcome operational barriers and streamline patient care. As Diabeter has established itself as an independent clinic, it may be valuable to consider the advantages and disadvantages of starting independently versus integrating into a hospital setting for future projects. Starting independently offers several strategic advantages, particularly when drawing parallels to the lean startup theory stating that transferring lean startup methods into larger organization have had limited success.^37^ Diabeter's independent model enables faster decision-making and greater flexibility in adapting to patient needs, fostering innovation in care delivery. However, this independence may also limit access to resources typically found in hospital environments. Conversely, integrating into a hospital could provide broader resources and established patient networks, enhancing credibility.

Crucially, **clear contractual agreements** should be defined to specify when and how patients will be referred between partners. Establishing these relationships early is important, but they also require ongoing attention and development throughout the implementation process. This idea is supported by recent findings that highlight Intermountain's strategic partnerships. Intermountain recognizes these collaborations as crucial for expanding its reach and enhancing its capacity to deliver VBC, emphasizing the importance of such partnerships for the future of healthcare.^38^

A focused strategy is essential. Organizations should identify and concentrate on **specific disease areas** or patient groups, tailoring services to deliver highly specialized, high-quality care. Additionally, **hybrid care models**—which combine telemedicine with in-person services—allow for more flexible and patient-centered care delivery, particularly in a post-pandemic world. Expanding geographic reach is crucial for scaling VBC initiatives. This could involve opening new facilities in **strategic locations.** Here it is crucial to concentrate on specific indications within diabetes management and invest in building a strong reputation for excellence in these areas (e.g. T2D, T1D).

### Enabling technology

#### Barriers

A lack of interoperable IT systems poses a significant barrier to achieving integrated care and measuring outcomes effectively. Without a unified IT infrastructure, organizations struggle with data fragmentation, inefficiency in communication, and the inability to track patient outcomes across various providers and services.

#### Facilitators (if not yet ready)

To enable integrated care and outcome measurement, organizations must invest in interoperable IT systems. This infrastructure should facilitate seamless communication and data sharing across various providers, departments, and external partners. A unified platform allows healthcare professionals to access complete patient information in real-time, ensuring that care decisions are well-informed and coordinated.

Organizations should adopt health IT solutions that enable outcome tracking and analytics. These systems should integrate clinical, financial, and operational data, enabling healthcare teams to track patient progress, identify patterns, and adjust treatment plans accordingly. Implementing decision-support tools within the IT platform can further assist physicians in delivering evidence-based care. Here, it needs to be ensured that the IT infrastructure is designed to connect with existing national health databases, EHRs, and other relevant systems, allowing for real-time data exchange and improved patient care.^33^

### Limitations and future directions

Our study faced several limitations that merit consideration. First, we did not interview an equal number of participants across all perspectives, which may have introduced bias. Regional differences within Switzerland may have also impacted the findings. For instance, in Western Switzerland, where integrated health projects like Résau de l’Arc already in place, participants may have been less critical of SwissDiabeter.^39^ This suggests that attitudes toward the project could vary depending on the local healthcare infrastructure and familiarity with similar initiatives.

Furthermore, the number of interviewees from the Netherlands and Switzerland was imbalanced. Although the focus was primarily on Switzerland to identify country-specific barriers, equal representation from both countries could have offered a balanced comparison of perspectives. The decision to prioritize Swiss participants was intentional, reflecting the study's aim to address specific challenges within the Swiss healthcare system.

Next, the study acknowledges potential biases in participant selection, particularly regarding vested interests in VBC adoption. To mitigate this, we included diverse stakeholders, including several skeptics of VBC. However, dissenting views may still be underrepresented, as most experts were engaged in the field. Future research could further explore perspectives from those less involved in VBC to provide a more balanced view.

Lastly, although Diabeter is considered in this study as a role model, showing superior health outcomes compared to other clinics, limited data is available on its specific cost structures and profitability mechanisms. As a result, any initiative, such as SwissDiabeter or similar projects in other countries aiming to replicate the Diabeter model, would need to conduct a thorough financial analysis to accurately assess the viability of the business case.

While the present study focuses on diabetes care in Switzerland, the insights related to bundled payment models, aligning incentives among providers, linking reimbursement to patient outcomes, and leveraging interoperable IT systems to track patient progress likely relate to other chronic conditions. Future research is needed to systematically apply and validate the identified facilitators across different disease areas and geographic contexts. For instance, the proposed VBC Readiness assessment framework could be adapted to oncology or cardiology to provide actionable strategies for advancing VBC-based models. Moreover, risk equalization mechanisms could further incentivize insurers to adopt bundled payment models and support VBC-oriented care models across different contexts. Future work could formally test and evaluate different risk equalization mechanisms across disease contexts.

## Conclusion

In summary, the SwissDiabeter initiative, modeled after the Diabeter clinic in the Netherlands, presents a promising advancement in today’s diabetes care. By integrating interoperable technology, SwissDiabeter helps address critical challenges such as the need for remote monitoring, personalized treatment plans and outcome-based care. However, the implementation of SwissDiabeter is not without challenges. Factors such as strong leadership, restructured financial incentives, digital infrastructure, and interdisciplinary collaboration are essential for its adoption and potential to deliver high quality and cost-effective care.

## Author's note

Elgar Fleisch is also affiliated with Centre for Digital Health Interventions (CDHI), Institute of Technology Management, University of St. Gallen, Switzerland. Tobias Kowatsch is also affiliated with Department of Management, Technology, and Economics, ETH Zurich, Switzerland.

## Appendix A

### Table A1: interview guideline for the founders of Diabeter Netherlands

**Table:**

1. Warm Up 8 Min / 00:02-00:10
Goal: Introduction into the topic and getting to know each other Tell me about you and your career and current position. 2. Treatment of insulin dependent patients 15 Min / 00:10-00:25
Goal: Understanding the current treament How do you currently perceive the care situation for insulin-dependent patients (chronically ill patients)? Have they reached their target yet? What do they find works well and less well in the Netherlands? How is your organization set up? How do the different specialists work together? (Exchange/Communication, Tools, Technology, etc.)? 3. Diabeter Model in Switzerland 10 Min / 00:25-00:35
Goal: Understanding the challenges and success factors What challenges did they (Diabeter) face in integrating your new concept into the existing healthcare system (specifically: hospitals, medical practices, health insurance companies, etc.)? (In your opinion), what are the success factors for a diabetes clinic like Diabeter to operate in Switzerland? What barriers are to be expected? 4. Financial models and Bundled Payments 10 Min / 00:35-00:45
Goal: Understanding how the business model could look like *Material: slides* How do you implement the concept of bundled payments? What do bundled payments include? What experience have you had with bundled payments? How is it received by the various stakeholder groups (in particular health insurance companies, patients, etc.)? What are the requirements for bundled payments to work with diabetes? Are there different bundled payments depending on the degree of illness? (Is a "sicker" patient paid with a higher bundled payment?) Are there other models that you could imagine or have considered? How severely did they restrict Dutch laws and how did they overcome these barriers? How do you see the potential of uninsured services? 5. Organization and processes 20 Min / 00:45-01:05
Goal: Understanding how they view Diabeter *Material: slides* How did they adapt incentive structures to encourage a focus on value to the patient? What structures or systems presented an barrier to a VBHC approach and needed to be overcome? How have you set up the organization within a center and how do employees need to be empowered so that they can reach their full potential and patients receive the best possible care? What was the attitude towards digitalization among employees? How do you evaluate the current status of digitalization at Diabeters? In which areas has it helped them? Where do you still see a potential gap? How have you designed the systems so that digitalization and data analysis can help support employees and patients? What partnerships do you think are important for a concept like Diabeter to work? How do the different HC-providers and partners need to work together to provide patient-centered care? How have you been able to make a change to more transparency in providing patient feedback? What challenges do you see in integrating patient feedback into the healthcare process to improve the patient experience? How can staff acceptance be ensured? How openly can this currently be handled? To what extent do you believe that there is a demand from patients and employees to make use of such a solution or to work at such a clinic? Which functions that are not found in a conventional hospital or medical center had to be created in order to implement a concep like Diabetert? 6. Wrap-Up 5 Min / 01:00-01:05
Is there anything we have not yet addressed in relation to the challenges of treating insulin-dependent patients? Is there anything else you would like to tell us about this topic? That brings us to the end of the interview. Thank you very much!

### Table A2: Interview guideline: patient associations

**Table:**

1. Warm Up 8 Min / 00:02-00:10
Ziel: Einstieg in das Gespräch. Auflockerung. Person etwas näher kennenlernen. Einschätzen können, wie erfahren die Person in Bezug auf insulinpflichtigen Diabetes ist. Was genau ist Ihre Funktion? Seit wie vielen Jahren sind Sie in dieser Funktion? Was ist ihre Rolle in der Begleitung und Unterstützung von Diabetes-Patienten? Wenn Sie an insulinpflichtigen Diabetes denken, was geht Ihnen da spontan durch den Kopf? Wir sprechen heute nur von insulinpflichtigen Patienten (d.h. i.d.R. Diabetes-Typ1-Patienten): Gibt es aus Ihrer Sicht Gruppen von Typ 1 und Typ 2 Patienten, die man voneinander unterscheiden müsste? Warum und bzgl. welchen Kriterien? *Stützen: Haben diese unterschiedliche Bedürfnisse?* 2. Behandlung von Diabetes Typ1 Patienten 20 Min / 00:10-00:30
Ziel: Verstehen, wie Patientenorganisationen die Behandlung von Diabetes-Typ 1 Patienten aktuell beurteilen. *Integration unter einem Dach (1 & 4)* Ich möchte nun mit Ihnen über die Behandlung von insulinpflichtigen Diabetes Patienten sprechen. Können Sie mir einen typischen Alltag eines Diabetes Patienten, der auf Insulin angewiesen ist, beschreiben? *Stützen: Was sind die grössten Herausforderungen im Alltag eines insulinpflichtigen Diabetes Patienten? Warum ist das so?* *Stützen: Und was funktioniert aus Ihrer Sicht heute gut?* **Was ist ihre Erfahrung: Gibt es einen Spezialisten (z.B. Diabetologe), der die verschiedenen Beteiligten koordiniert oder muss das der Patient selbst machen?** Wie ist es denn, wenn der Patient einen dieser Beteiligten besucht: Wie gut sind diese auf die Anliegen vorbereitet? **Sind wichtige Dokumente und Daten vorhanden und werden diese genutzt? (Daten, Berichte, Informationen zum Patienten)?** *Stützen: wie sind die Daten verfügbar (digital, Papier, CD-ROM, etc.)?* *Anreise (5)* Wie oft pro Jahr müssen die Patienten vor Ort vorbeigehen? Wie beurteilen Sie diese Frequenz? Wie beurteilen Sie die Anreise für die Patienten? Warum? Wie ist die Verfügbarkeit der Beteiligten? D.h. wie lange warten die Patienten, bis sie einen Termin erhalten? Gibt es sonst noch Hindernisse in Bezug auf die Konsultationen der Patienten bei den Spezialisten? *Z.B. Parkplätze?* **Kennen Sie Möglichkeiten, um die besprochenen Schwierigkeiten zu vermeiden und eine bessere Patient Journey zu garantieren? Welche?** Was ist Ihre Erfahrung: Wer unterstützt die Patienten sonst noch? (Familie, andere Betroffene, Patientenorganisation, Online-Communities etc.) Wie werden diese Personen / Organisationen einbezogen? Welche Schwierigkeiten treten dabei auf? *Stützen: Welche Herausforderungen gibt es für Eltern bei der Betreuung ihrer Kinder / Jugendlichen?* 3. Daten in der Behandlung von insulinpflichtigen Diabetes Patienten 10 Min / 00:30-00:40
Ziel: Verstehen, wie Daten in der Behandlung verwendet werden und welchen Nutzen sie aus Sicht der Befragten bringen. *Ergebnisse messen (2) Data Sharing & Telemedizin (6)* Inwiefern werden heute die Messwerte der Patienten gemessen und beobachtet? Welche Schwierigkeiten gibt es dabei? Welche Rolle spielen Apps / neue Technologien für den Patienten und sein Betreuungsteam? Wie stark ist die Patient Journey schon digitalisiert? **Wie beurteilen Sie die Bereitschaft von Patienten für die Nutzung von Apps und neuen Technologien? Warum?** Gibt es Patienten, die mit ihren Betreuungsteam über Telemedizin verbunden sind? Wie beurteilen Sie die Einbindung von Telemedizin? Warum? 4. Bezahlung 5 Min / 00:40-00:45
*Bereitschaft mehr zu zahlen e.g. Zusatzversicherung (3)* Inwiefern funktioniert heute die Bezahlung der Behandlung? Wird alles von der Grundversicherung abgenommen? Gibt es Leistungen, welche von der Zusatzversicherung beansprucht werden? Was müssen Patienten selbst bezahlen? Gibt es von den Patienten Wünsche zu einem anderen Tarif-System oder einer Zusatzversicherung für chronisch Kranke? Gibt es Dinge die sie ändern würden? 5. SwissDiabeter 10 Min / 00:45-00:55
Ziel: Verstehen, wie das Konzept von SwissDiabeter beurteilt wird. *Material: Folien mit Visualisierung des Konzepts* Konzept Swiss Diabeter vorstellen (allenfalls Bezug nehmen auf das Modell in den Niederlanden) Aktuell untersuchen wir alternative Versorgungsmodelle im Bereich von Diabetes und wie solche Modelle in der Schweiz umgesetzt werden können. Insbesondere schauen wir uns die Niederlande mit ihrer Klink-Kette «Diabeter» an. Dabei geht es darum mittels eines Value-based healthcareansatzes den Patienten für sie relevante Verbesserungen ihrer Gesundheit mit einem besseren Erlebnis ihrer Versorgung bereit zu stellen. Da diese Klinik sehr erfolgreich ist, möchten wir prüfen, ob eine solche auch für die Schweiz interessant sein könnte. **Wie würde eine Diabetes Klinik ähnlich wie Diabeter ins Versorgungsnetz der Schweiz reinpassen? Wo denken Sie, gäbe es Schwierigkeiten bei der Implementierung?** Solch eine Diabetes Klinik würde es nur an wenigen ausgewählten Standorten in der Schweiz geben. Wie weit wären Patienten bereit, in eine solche Diabetes Klinik zu reisen? Wie lange reisen sie heute schon, um ihren Arzt zu sehen? Eine integrierte Diabetes Klinik würde den Patienten Leistungen, wie Trendüberwachung ihrer Werte, Konsultationen, über telemedizinische Kanäle anbieten. Sind aus ihrer Sicht Patienten bereit ihre Werte automatisiert mit ihrem Betreuungsteam zu teilen? Sind sie bereit bis auf eine jährliche vor-Ort Kontrolle, sofern ihre übermittelten Werte im Zielbereich liegen, den Kontakt telemedizinisch zu führen? Inwiefern fehlt es den Patienten aktuell am Austausch mit anderen Diabetes Patienten (peer support)? Sehen Sie einen Nutzen in einem verstärkten Patientenaustausch? 6. Wrap-Up 5 Min / 00:55-01:00
Gibt es etwas, das wir in Bezug auf die Herausforderungen in der Behandlung von insulin-abhängigen Patienten bis jetzt noch nicht angesprochen haben? Möchten Sie uns zu diesem Thema noch etwas mit auf den Weg geben? Damit wären wir am Ende der Befragung. Herzlichen Dank!

### Table A3: Interview guideline: health insurances

**Table:**

1. Warm Up 8 Min / 00:02-00:10
Ziel: Einstieg in das Gespräch. Auflockerung. Person etwas näher kennenlernen. Einschätzen können, wie erfahren die Person in Bezug auf Diabetes Typ1 ist *Material: -* Was genau ist Ihre Funktion? Seit wie vielen Jahren sind Sie in dieser Funktion? Wie stark beschäftigen Sie sich mit dem Gesundheitssystem Schweiz? Was wissen Sie über das Thema Diabetes (insbesondere Typ 1)? *Falls nicht viel, auf chronische Krankheiten wechseln* 2. Behandlung von Diabetes Typ1 Patienten 20 Min / 00:10-00:30
Ziel: Verstehen, wie Versicherungen die Behandlung von insulinabhängigen Patienten aktuell beurteilen. Ich möchte nun mit Ihnen über die Behandlung von insulinpflichtigen (chronisch Kranken) Patienten sprechen. Wie nehmen Sie das Behandlungssystem von insulinpflichtigen Patienten in der Schweiz wahr? *Stützen: Wo sehen Sie Schwächen? Wo Stärken?* Die Behandlung von Diabetes Typ 1 Patienten ist sehr kostspielig. Die Zahl der Diabeteserkrankten und allgemein chronisch Kranken in der Schweiz nimmt stetig prozentual zu. Was für eine Bedeutung hat es, dass immer mehr Leute Diabetes haben? Was hat das für eine Auswirkung auf die Prämien? Wie könnte man die hohen Kosten der Behandlung von Diabetes Patienten verringern? Inwiefern werden von der Krankenkasse Kliniken und Spitäler bei der Behandlung von Typ 1 Diabetes miteinander verglichen? (z.B. Kosten pro Patienten) Was fehlt in der Grundversicherung? Gibt es auch noch Dinge, die in der Zusatzversicherung fehlen? Muss man sich noch andere Versicherungsmodelle überlegen? *(Stützpunkt: Multimed CSS)* 3. SwissDiabeter 25 Min / 00:30-00:55
Ziel: Verstehen, wie das Konzept von SwissDiabeters beurteilt wird. *Material: Folien mit Visualisierung des Konzepts* Konzept Swiss Diabeter vorstellen (allenfalls Bezug nehmen auf das Modell in den Niederlanden) Aktuell untersuchen wir alternative Versorgungsmodelle im Bereich von Diabetes und wie solche Modelle in der Schweiz umgesetzt werden können. Insbesondere schauen wir uns die Niederlande mit ihrer Klink-Kette «Diabeter» an. Dabei geht es darum mittels eines Value-based healthcare Ansatzes qualitativere und gleichzeitig günstigere Behandlungen für Patienten bereit zu stellen. Da diese Klinik sehr erfolgreich ist, möchten wir prüfen, ob eine solche auch für die Schweiz interessant sein könnte. Was denken Sie über das Konzept von Diabeter? Wie würde eine Diabetes Klinik ähnlich wie Diabeter ins Versorgungsnetz der Schweiz reinpassen? Wo denken Sie, gäbe es Schwierigkeiten bei der Implementierung? Unter welchen Voraussetzungen könnte dieses System in der Schweiz funktionieren? Worin sehen Sie in einer Diabetes Klinik den Nutzen für Ihre versicherten Patienten in der Schweiz? Beim value-based healthcare System wird das Vergütungsmodell «Bundled Payments» verwendet. Wie stehen Sie zu bundled payments? Was sind die Voraussetzungen dafür, dass bundled payments bei einer Diabetes Erkrankung funktionieren? Was für Anreize gäbe es bei einem solchen Modell für Versicherungen? (z.B. Verfeinerung Risikoausgleich) Gibt es andere Zahlungsmodelle, die Sie sich vorstellen könnten? Wie stark schränken die aktuellen Gesetze (KVG) einen ein, um solche VBHC basierten Modelle in der Schweiz umzusetzen? 4. Wrap-Up 5 Min / 00:55-01:00
Gibt es etwas, das wir in Bezug auf die Herausforderungen in der Behandlung von insulinpflichtigen Patienten bis jetzt noch nicht angesprochen haben? Möchten Sie uns zu diesem Thema noch etwas mit auf den Weg geben? Damit wären wir am Ende der Befragung. Herzlichen Dank!

### Table A4: Interview guideline: healthcare providers

**Table:**

1. Warm Up 8 Min / 00:02-00:10
Ziel: Einstieg in das Gespräch. Auflockerung. Person etwas näher kennenlernen. Einschätzen können, wie erfahren die Person in Bezug auf insulinpflichtigen Diabetes ist Was genau ist Ihre Funktion? Seit wie vielen Jahren sind Sie in dieser Funktion? Haben Sie ein Spezialgebiet? Wie sieht ihr Alltag bei der Betreuung von Patient:innen aus? Wenn Sie an insulinpflichtigen Diabetes denken, was geht Ihnen da spontan durch den Kopf? Wir sprechen heute nur von insulinpflichtigen Patienten (d.h. i.d.R. Diabetes-Typ1-Patienten): Gibt es aus Ihrer Sicht Gruppen von Typ 1 und Typ 2 Patienten, die man voneinander unterscheiden müsste? Warum und bzgl. welchen Kriterien? *Stützen: Haben diese unterschiedliche Bedürfnisse?* 2. Behandlung von insulinpflichtigen Diabetes Patienten 10 Min / 00:10-00:20
Ziel: Verstehen, wie die Patient:innen aktuell versorgt werden Überprüfen von folgenden Hypothesen: Personal hat wenig Zeit / Patienten werden nicht rundum betreut / aktuelles System stosst an ihre Grenzen Ich möchte nun mit Ihnen über die Behandlung von insulinpflichtigen Diabetes Patienten sprechen. Wie schätzen sie die aktuelle Betreuung von Diabetes Typ-1 Patient:innen in der Schweiz ein? Welche Herausforderungen sehen Sie im aktuellen Gesundheitssystem, die die optimale Betreuung von Patienten mit Diabetes Typ 1 beeinträchtigen könnten? Wo sehen sie die Stärken Welche Herausforderungen stellen sich aktuell für ihre Klinik/Praxis, um Patient:innen umfangreich zu behandeln und diese im Alltag zu unterstützen? Welche Faktoren beschränken ihre Kapazitäten? Wie sehen sie die aktuelle Dauer der Behandlung? Inwiefern fehlt ihnen eine Spezialisierung für nur Diabetes 1 Patient:innen? Wie werden Behandlungserfolge bisher gemessen? (wenn überhaupt) 3. Organisation 15 Min / 00:20-00:35
Ziel: Verstehen, wie die Struktur o. Organisation der Praxen o. Kliniken aussieht. Überprüfen von folgenden Hypothesen: Patienten werden in Silos versorgt (chaotisch) / Zusammenarbeit nicht effizient / Angehörige wissen nicht wie unterstützen / Digitalisierung nicht fortgeschritten (1) *Integration unter einem Dach (4) Zusammenarbeit* Wie empfinden Sie aktuell die Zusammenarbeit zwischen den verschiedenen Fachbereichen? Wodurch werden Schwierigkeiten bei der Koordination der Pflege von Diabetes Typ 1-Patienten zwischen verschiedenen Fachbereichen (Ärzte, Pflege, Therapeuten, Ernährungsberater, etc.) erzeugt? Inwiefern beziehen Sie nahstehende Familienmitglieder in die Behandlung mit ein? Inwiefern unterstützen Familie und Online Communities den Diabetiker? Nehmen Sie bei ihrer Arbeit fehlendes Vertrauen einer Funktion gegenüber einer anderen Funktion wahr, welche die optimale Versorgung verhindert?*Beispiel: Ärzte vertrauen Pflegefachpersonen nicht* (2) *Ergebnisse messen (6) Data Sharing & Telemedizin* Inwiefern arbeiten Sie heute mit Technologie? Welche Tools benutzen Sie für was? Welche Vor- und Nachteile sehen Sie bei der zunehmenden Digitalisierung? (Stütze: Telemedizin, Austausch zwischen Ärzten, Ärzte-Patienten-Austausch) Wie stehen Sie allgemein zur weiteren Digitalisierung? Wie werden Behandlungserfolge bisher gemessen? (wenn überhaupt)? 4. SwissDiabeter 30 Min / 00:35-01:05
Ziel: Verstehen, wie das Konzept von SwissDiabeter beurteilt wird. *Material: Folien mit Visualisierung des Konzepts* Konzept Diabeter vorstellen (auch wenn schon bekannt, kurzes Wrap-up, damit man vom gleichen spricht) (1) *Integration unter einem Dach (4) Zusammenarbeit (5) Standort* Kennen Sie Diabeter aus den Niederlanden schon? Was geht Ihnen spontan durch den Kopf? Welche Vor- und Nachteile könnte eine Spezialisierung im ambulanten Bereich (Centers) mit sich bringen? Könnten Sie sich vorstellen, dass man so etwas in der Schweiz implementieren kann? Sehen Sie für die Schweiz speziell Hürden oder Erleichterungen für die Implementierung bei einer solchen Klinik? Wie muss die Organisation innerhalb eines Centers aussehen bzw. wie müssen Mitarbeitende befähigt werden, damit das Potenzial der Mitarbeitenden voll ausgeschöpft werden kann und die Patienten die bestmögliche Betreuung erhalten? Welche Tätigkeiten könnten auch von spezialisierten Pflegefachpersonen ausgeführt werden, welche aktuell von Ärzt:innen übernommen wird? Inwiefern kann die Remote-Behandlung ihnen helfen, ihre Kapazitäten besser zu nutzen? (nur nötige Termine anstatt Regular Check-ups) Solch eine Diabetes Klinik würde es nur an wenigen ausgewählten Standorten in der Schweiz geben. Wie weit wären **ihre** Patienten bereit, in eine solche Diabetes Klinik zu reisen, unter Berücksichtigung, dass sie auch Telemedizinisch betreut werden würden? Könnten Sie (als Diabetologe, Fachpersonal, etc.) sich vorstellen, in einer Diabetes Klinik zu arbeiten? Unter welchen Voraussetzungen? (2) *Ergebnisse messen (6) Data Sharing & Telemedizin* Welche bürokratischen Hindernisse müssen beseitigt werden, damit sie sich auf ihre Arbeit fokussieren können? Inwiefern kann Ihnen dabei die Digitalisierung helfen? Inwiefern können innovative Technologien (z.B. Datentracking von Blutwerten mit intelligenten Analysen) sie bei der Arbeit unterstützen? Welche Faktoren spielen eine Rolle, damit Sie dem System vertrauen und nutzen? Inwiefern Wie transparent wird mit Patientenfeedback umgegangen? Welche Herausforderungen sehen Sie bei der Integration von Patientenfeedback in den Gesundheitsversorgungsprozess, um die Patientenerfahrung zu verbessern? Inwiefern kann die neu gewonnene Transparenz über die "Performance" von Ärzten und Kliniken die Behandlung verbessern? Wo sehen Sie Vor - und Nachteile von mehr Transparenz 5. Wrap-Up 5 Min / 01:05-01:10
Gibt es etwas, das wir in Bezug auf die Herausforderungen in der Behandlung von insulin-abhängigen Patienten bis jetzt noch nicht angesprochen haben? Möchten Sie uns zu diesem Thema noch etwas mit auf den Weg geben? Damit wären wir am Ende der Befragung. Herzlichen Dank!

## Appendix B

**Table B1.** Consolidated criteria for reporting qualitative studies (COREQ): 32-item checklist Developed from Tong et al.^23^

No. item	Guide questions/description	Reporting
**Domain 1: Research team and reﬂexivity**		
**Personal Characteristics**		
1. Interviewer/facilitator	Which author/s conducted the interview or focus group?	– Odile-Florence Giger
2. Credentials	What were the researcher's credentials? *E.g. PhD, MD*	– Odile-Florence Giger: M.Sc., B.Sc. – Mia Jovanova: PhD – Tobias Kowatsch: Prof. – Elgar Fleisch: Prof.
3. Occupation	What was their occupation at the time of the study?	– Odile-Florence Giger : Research assistant – Mia Jovanova: Research assistant – Tobias Kowatsch: Postdoctoral researcher – Elgar Fleisch: Professor
4. Gender	Was the researcher male or female?	– Odile-Florence Giger: female – Mia Jovanova: female – Tobias Kowatsch: male – Elgar Fleisch: male
5. Experience and training	What experience or training did the researcher have?	All research team members who conducted the interviews have experience to conduct qualitative research.
**Relationship with participants**		
6. Relationship established	Was a relationship established prior to study commencement?	The researcher that conducted the interviews had no prior relationships with the participants before the interviews took place. As a result, the interviewers should be considered unbiased.
7. Participant knowledge of the interviewer	What did the participants know about the researcher? *E.g. personal goals, reasons for doing the research*	The objective of this study was communicated through e-mail. Moreover, the objectives of the study and research purpose were mentioned at the outset of each individual interview.
8. Interviewer characteristics	What characteristics were reported about the interviewer/ facilitator? *E.g. bias, assumptions, reasons, and interests in the research topic*	Information regarding the interviewers’ characteristics and their professional backgrounds was provided on the institute's website. At the commencement of each interview, during the introduction phase, interviewees were informed of the interviewers’ educational backgrounds and current occupations.
**Domain 2: Study design**		
**Theoretical framework**		
9. Methodological orientation and theory	What methodological orientation was stated to underpin the study? *E.g. grounded theory, discourse analysis, ethnography, phenomenology, content analysis*	The study was based on a qualitative research methodology conducting semi-structured interviews. We used inductive thematic analysis (please refer to the “Methods” section, p. 6 in manuscript).
**Participant selection**		
10. Sampling	How were participants selected? *E.g. purposive, convenience, consecutive, snowball*	Participant recruitment and selection were carried out using a combination of diverse sampling approaches. Purposive sampling was employed to encompass a wide spectrum of expertise and functions. Additionally, the snowballing technique was utilized to enhance diversity across demographics, and experiences.
11. Method of approach	How were participants approached? *E.g. face-to-face, telephone, mail, email*	We approached the participants via e-mail. Most of the semi-structured interviews were conducted via video calls, while a portion were in-person.
12. Sample size	How many participants were in the study?	A total of 27 participants took part in the study (see p. 4 in manuscript).
13. Non-participation	How many people refused to participate or dropped out? Reasons?	A total of 11 participants discontinued their involvement in the study. Reasons for dropping out were time constraints or being unreachable after initially agreeing to participate.
**Setting**		
14. Setting of data collection	Where was the data collected? *E.g. home, clinic, workplace*	The interviews were conducted online or at the workplace of the participant.
15. Presence of non-participants	Was anyone else present besides the participants and researchers?	No other individuals were present.
16. Description of sample	What are the important characteristics of the sample? *E.g. demographic data, date*	For specific details about the participants’ professional attributes, please refer see Table C1 in Appendix C, pp. 1–2.
**Data collection**		
17. Interview guide	Were questions, prompts, guides provided by the authors? Was it pilot tested?	The interview guideline was pilot-tested before the first interview. The participants were informed that no preparation was needed before the interview. The overall interview flow and agenda were explained during the personal introduction. (see p. 5 in manuscript)
18. Repeat interviews	Were repeat interviews carried out? If yes, how many?	None of the interviews were repeated.
19. Audio/visual recording	Did the research use audio or visual recording to collect the data?	All interviews were recorded in audio format following the participants’ consent (see p. 6 in manuscript).
20. Field notes	Were ﬁeld notes made during and/or after the interview or focus group?	No field notes were taken.
21. Duration	What was the duration of the interviews or focus group?	The interviews had an average duration of 69 minutes.
22. Data saturation	Was data saturation discussed?	The research team consistently discussed data saturation (see p. 6 in manuscript).
23. Transcripts returned	Were transcripts returned to participants for comment and/or correction?	Interview transcripts were provided to the participants for comment and corrections. Subsequent modifications were made based on their input and feedback. If no response or feedback to the transcript was received within a week, it was regarded as acceptable.
**Domain 3: analysis and ﬁndings**		
**Data analysis**		
24. Number of data coders	How many data coders coded the data?	Data coding was performed by three people (see p. 6 in manuscript).
25. Description of the coding tree	Did authors provide a description of the coding tree?	We have created list of codes and hierarchies of the thematic analysis of the interview data.
26. Derivation of themes	Were themes identiﬁed in advance or derived from the data?	Thematic analysis was employed to identify, analyze, and present first-order concepts, second-order themes, and aggregated dimensions that emerged from the data (see p. 6 in manuscript).
27. Software	What software, if applicable, was used to manage the data?	For data analysis and management, “ATLAS.ti” software was used (see p. 6 in manuscript).
28. Participant checking	Did participants provide feedback on the ﬁndings?	Aggregated dimensions were presented and discussed with two study participants in an online meeting.
**Reporting**		
29. Quotations presented	Were participant quotations presented to illustrate the themes/ﬁndings? Was each quotation identiﬁed? *E.g. participant number*	Participant quotations were presented to illustrate themes and findings. Each quotation is attributed to a participant number ensuring clear identification by the authors (see pp. 8 −15 in manuscript)
30. Data and ﬁndings consistent	Was there consistency between the data presented and the ﬁndings?	Yes, there was consistency between the data presented and the findings.
31. Clarity of major themes	Were major themes clearly presented in the ﬁndings?	Yes, the major results were clearly presented in the findings, please refer to “Results” (see pp. 8–14 in manuscript).
32. Clarity of minor themes	Is There A Description Of Diverse Cases Or Discussion Of Minor Themes?	Yes, There Is A Description Of Diverse Cases And A Discussion Of Minor Themes, Please Refer To “Results” And “Discussion” (see pp. 8–16 in manuscript).

## Appendix C

**Table C1.** Interview participants.

Participant	Perspective	Interviewees background	Years of experience	Duration	Country
Interviewee 1	Provider (Physician)	Endocrinologist and diabetologist	25 +	73 minutes	Switzerland
Interviewee 2	Provider (Physician)	Clinic director endocrinology and diabetology	25 +	65 minutes	Switzerland
Interviewee 3	Provider (Physician)	Clinic director endocrinology and diabetology	25 +	48 minutes	Switzerland
Interviewee 4	Provider (Healthcare executive and physician)	Chief physician telemedicine company	15 +	61 minutes	Switzerland
Interviewee 5	Provider (Physician)	Clinic director endocrinology and diabetology	20 +	35 minutes	Switzerland
Interviewee 6	Provider (Physician)	Clinic director endocrinology and diabetology	15 +	55 minutes	Switzerland
Interviewee 7	Provider (Healthcare executive and physician)	Founder Diabeter	30 +	66 minutes	Netherlands
Interviewee 8	Provider (Healthcare executive and physician)	Founder Diabeter	30 +	64 minutes	Netherlands
Interviewee 9	Provider (Healthcare executive)	CEO of a hospital	10 +	30 minutes	Switzerland
Interviewee 10	Provider (Physician)	Clinic manager endocrinology and diabetology	15 +	55 minutes	Switzerland
Interviewee 11	Patient Organization	Diabetes society	15 +	64 minutes	Switzerland
Interviewee 12	Patient Organization	Diabetes patient organization and nutritionist	10 +	58 minutes	Switzerland
Interviewee 13	Patient Organization	Diabetes consultant	20 +	49 minutes	Switzerland
Interviewee 14	Patient Organization	Diabetes organization	15 +	63 minutes	Switzerland
Interviewee 15	Patient Organization	Diabetes center	20 +	66 minutes	Switzerland
Interviewee 16	Payer	Insurance association	15 +	56 minutes	Switzerland
Interviewee 17	Payer	Pricing health insurance	15+	58 minutes	Switzerland
Interviewee 18	Payer	Sales, health insurance	20 +	55 minutes	Switzerland
Interviewee 19	Payer	Board member health insurance	30+	45 minutes	Switzerland
Interviewee 20	Payer	Division manager, health insurance	25+	67 minutes	Switzerland
Interviewee 21	Payer	CEO, Health insurance	20+	47 minutes	Netherlands
Interviewee 22	Payer	CEO, Health insurance	20+	46 minutes	Switzerland
Interviewee 23	Provider	Innovation manager	15 +	55 minutes	Switzerland
Interviewee 24	Payer	Innovation manager, Health insurance	20 +	58 minutes	Switzerland
Interviewee 25	Provider	CEO, clinic	20 +	44 minutes	Netherlands
Interviewee 26	Payer	Innovation manager, Health insurance	20 +	85 minutes	Switzerland
Interviewee 27	Payer	Innovation manager, Health insurance	10 +	85 minutes	Switzerland
