# Corrigendum to “Barriers and facilitators of implementing value-based care: The case of SwissDiabeter”

**DOI:** 10.1177/20552076261470160

**Published:** 2026-07-28

Giger O-F, Fleisch E, Kowatsch T, Jovanova M. Barriers and facilitators of implementing value-based care: The case of SwissDiabeter. *Digit Health*. 2025;11. doi:10.1177/20552076251336322.

In the above-mentioned article, the author order was listed as “Odile-Florence Giger, Elgar Fleisch, Mia Jovanova and Tobias Kowatsch”. The author order in the byline has been revised and should be read as:

Odile-Florence Giger, Elgar Fleisch, Tobias Kowatsch and Mia Jovanova

Additionally, Tobias Kowatsch and Mia Jovanova’s equal contributions as the senior authors have been added.

The article has been updated to reflect the corrections.

